# Extraskeletal Ewing's Sarcoma With Vertebral Metastasis: A Case Report

**DOI:** 10.7759/cureus.30878

**Published:** 2022-10-30

**Authors:** Tharamel M Muhuesein, Gurubharath Ilangovan, Alex Daniel Prabhu Arul Pitchai, Ealai A Parthasarathy, Rajamani Anand, Alam Khalil-Khan

**Affiliations:** 1 Radiology, Chettinad Hospital and Research Institute, Chettinad Academy of Research and Education, Chennai, IND; 2 Family Medicine, University of Sheffield, Sheffield, GBR

**Keywords:** case report, paravertebral mass, small round blue cell tumor, soft tissue neoplasms, extra skeletal ewing’s sarcoma

## Abstract

Ewing's sarcoma (ES) is the second most common osseous tumor in young patients after osteosarcoma. All primitive neuroectodermal tumors (PNET) and Askin tumors are members of Ewing's sarcoma family of tumors (ESFT), which all have aberrant translocations between the 11th and 22nd chromosomes. Only one in five cases of Ewing's sarcoma occurs as extraskeletal. In this report, we describe a young female with a palpable lump on her spine who presented with paravertebral and thoracic extraskeletal Ewing's sarcoma (EES). Over six months, the swelling gradually increased in size, and the patient reported episodes of episodic pain and fever. Examining the swelling, a non-reducible, non-tender ovoid lump measuring approximately 8 cm x 5 cm was found to have smooth margins and be slightly mobile. The use of magnetic resonance imaging (MRI) helped diagnose, plan surgical resections, assess neoadjuvant chemotherapy effectiveness, and detect local recurrences and metastatic spread of the tumor. The differential diagnosis of EES included embryonal rhabdomyosarcoma and lymphoma. The use of immunohistochemical markers further differentiated the diagnoses. In conclusion, it should be noted that EES, though rare, should be considered when evaluating soft tissue lumps of neoplastic characteristics, in children or adolescents. Considering the poor prognosis of this disease, early detection is essential. The MRI plays a vital role in diagnosing cancer, staging it locally, assessing response to neoadjuvant therapy, and identifying local recurrences and metastases.

## Introduction

Ewing's sarcoma of bone (ESB), extraskeletal Ewing's sarcoma (EES), primitive neuroectodermal tumors (PNET), and Askin tumor are all members of the Ewing's sarcoma family of tumors (ESFT) [1]. The most frequent and aggressive malignant bone and soft tissue tumor in children and adolescents is Ewing's sarcoma, which ranked behind multiple myeloma, osteosarcoma, and chondrosarcoma. Ninety-five percent of cases occur between the ages of four and 25 [2], with a peak incidence in the first and second decades. Males are 1.5 times more likely to be affected than females. Usually, Ewing's sarcoma affects proximal long bones at diaphysis, especially in the lower extremities. In 21% of cases, the femur is affected. Furthermore, it may affect bones in the pelvis and vertebrae as well as soft tissues on rare occasions [3]. In contrast to Ewing's sarcoma, extraosseous Ewing's sarcoma affects a slightly older age group (by five to 10 years), with the onset occurring in the second decade of life. Unlike classic skeletal variants, there is no distinct gender predilection, and the disease affects the trunk more centrally (rather than the extremities). Patients commonly seek consultation for pain and swelling over the affected area. Even though most patients present with a local disease process, they almost always have undetected metastatic spread. As a result of the localized disease process, the five-year survival rate is greater than that of later disease stages. Recent advances in chemotherapy have improved the survival rate [4]. To improve patient outcomes, the lesion must be detected early on imaging.

## Case presentation

**Figure 1:**
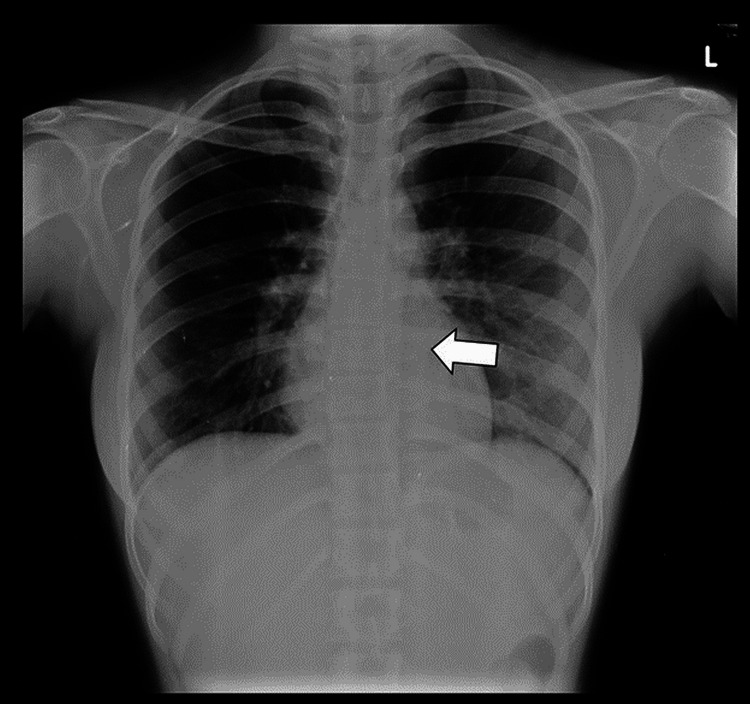
Chest X-ray (PA view) The X-ray shows a mild widening of the left paravertebral stripe in the lower thoracic region (arrow) signifying soft tissue involvement. No significant lung parenchymal or rib involvement was evident. PA: Posteroanterior

A 16-year-old female with no significant family history presented with swelling in her back that developed gradually over six months. In addition to the swelling, the patient reported pain and episodes of fever as the swelling grew. There was no discharge associated with the swelling. When palpated, a non-reducible, non-tender, ovoid hard lump measuring approximately 8 cm x 5 cm with smooth margins was noted. During the examination, the lump was located in the left dorsal paraspinal region and was not warm to touch. A chest X-ray demonstrated a mild enlargement of the left paravertebral space in the lower thoracic region, which suggested soft tissue involvement, as shown in Figure 1. 

**Figure 2:**
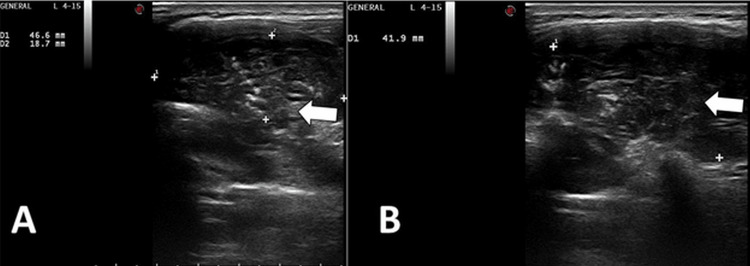
Ultrasound scan of the lump Axial (A) and sagittal (B) view shows a heterogeneously hypoechoic lesion (arrow) involving soft tissue and left paravertebral muscles with deeper extension into posterior intervertebral space.

A heterogeneously hypoechoic mass with anechoic areas in the left paravertebral region extending into the thoracic cavity posteriorly was detected by ultrasound (Figure 2).

**Figure 3:**
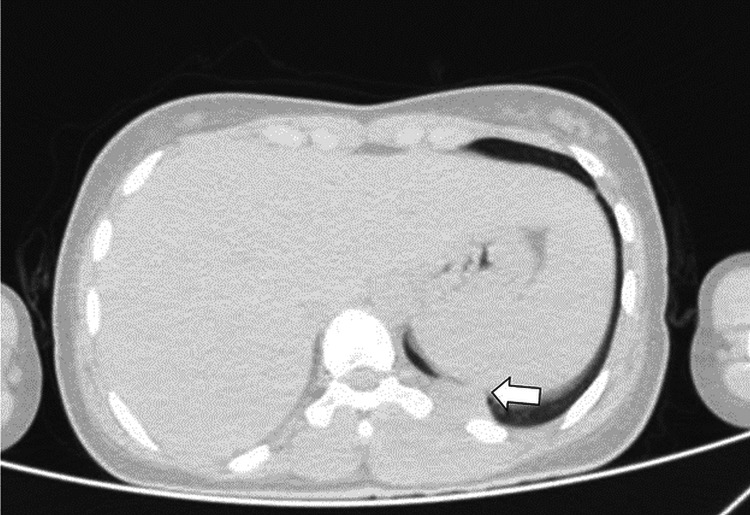
CT scan of the mass Axial non-contrast CT shows a mass (arrow) arising from paravertebral muscles and thoracic soft tissue with involvement of the left pleura. Note that there is no rib erosion.

Computed tomography (CT) scans were performed to further accurately evaluate the thorax, the vertebrae, the lung parenchyma, and the mediastinum and pleura. A large, well-defined heterogenous mass was noted from the 9th to the 12th dorsal vertebral spine, which was isodense to muscle. The CT scan confirmed no rib erosions nor any involvement of the lung parenchyma. It is apparent, however, that the left pleura was involved without significant pleural effusion as shown in Figure 3. 

**Figure 4:**
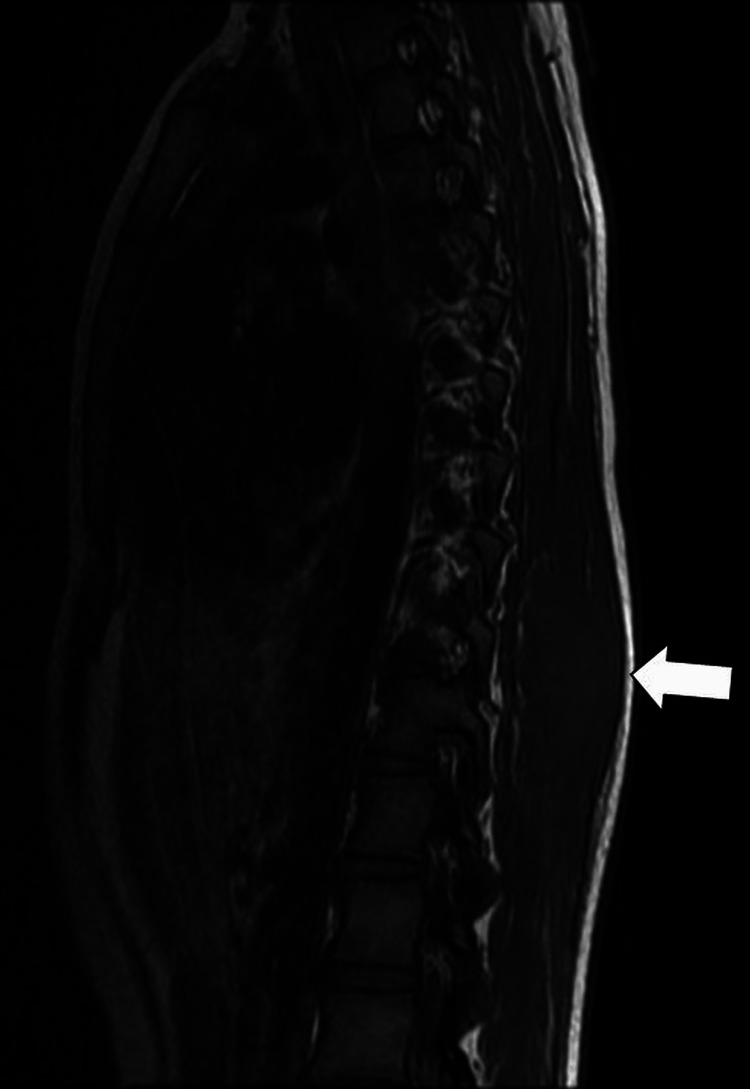
Saggital T1-weighted MRI shows a lesion (arrow) that has low to intermediate signal intensity and isointense to muscle when it extends from the D9 to the D12 vertebrae.

**Figure 5:**
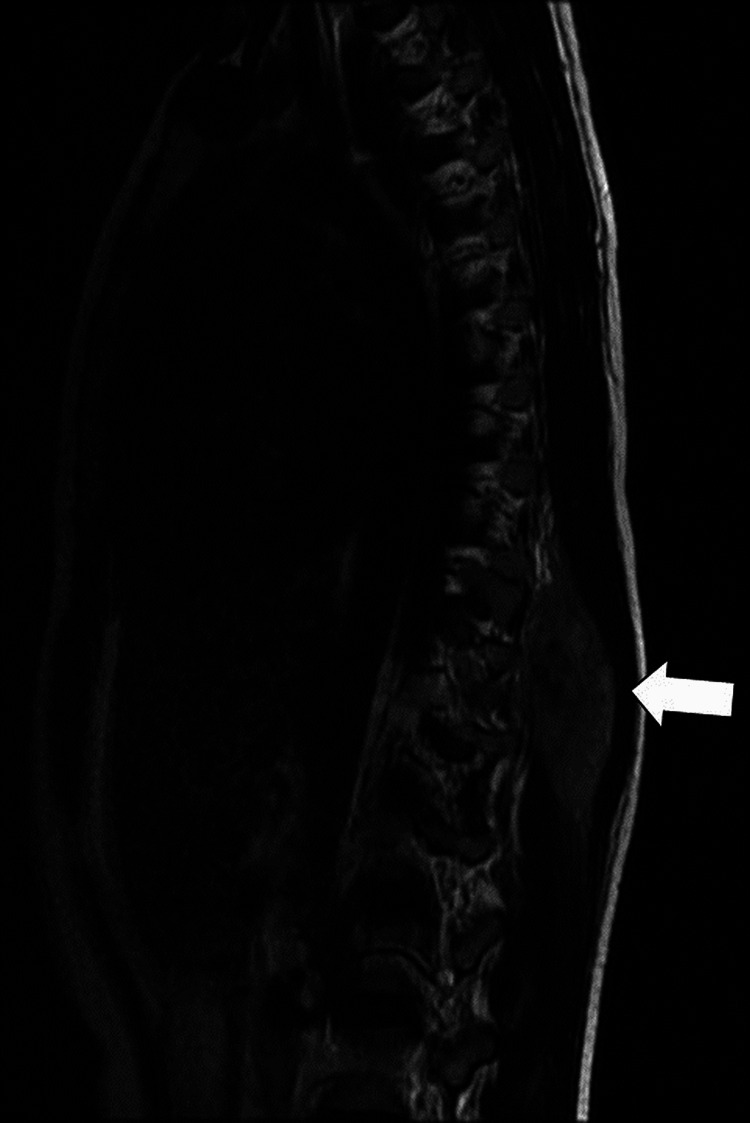
Saggital T2-weighted MRI shows a lesion (arrow) that has heterogeneously high signal intensity.

**Figure 6:**
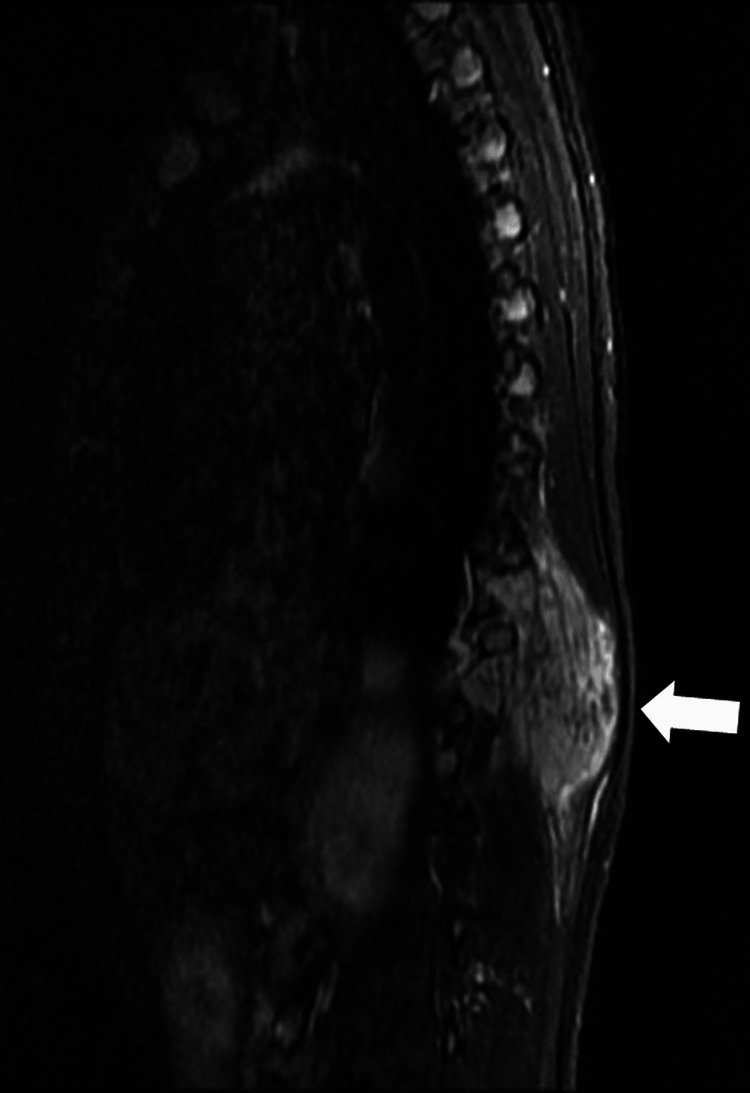
Saggital T1-weighted MRI post-contrast shows an intensely enhanced lesion (arrow).

Contrast MRI revealed a well-defined, lobulated T1 hypointense (Figure 4) and T2 heterogenous (Figure 5) intensity mass extending from D9 to D12 vertebrae with intense contrast enhancement (Figure 6).

**Figure 7:**
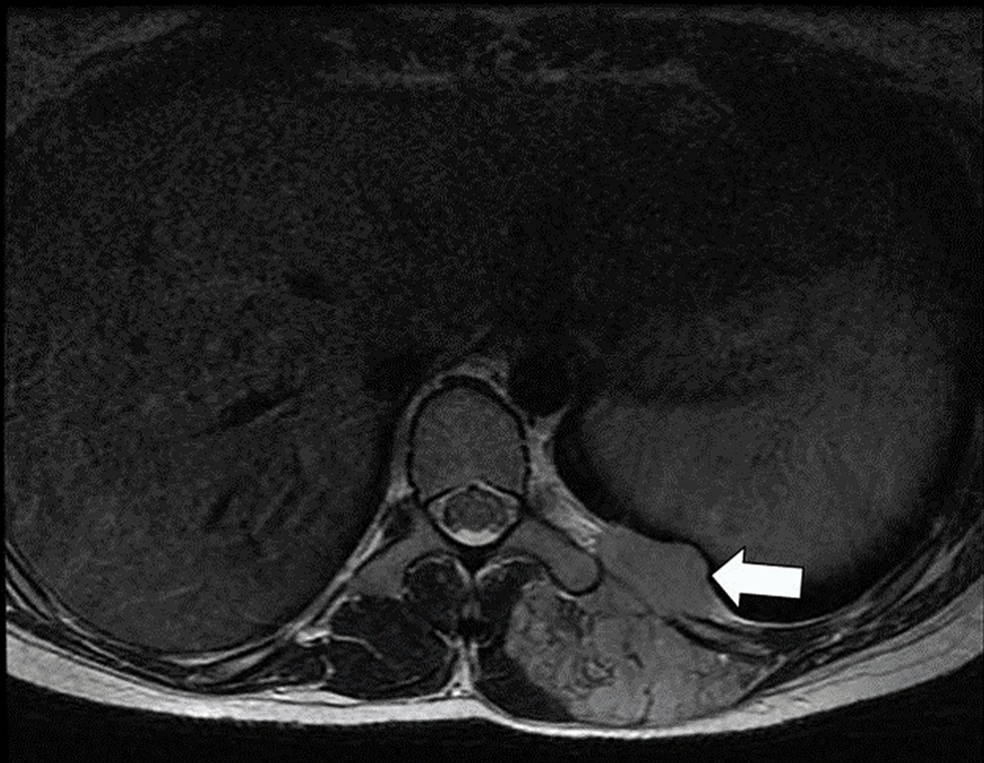
Axial T2-weighted MRI reveals a left paravertebral mass involving the left pleura (arrow).

As demonstrated in Figure 7, a mass involving the left paravertebral muscles, thoracic soft tissue, and left pleura was observed.

**Figure 8:**
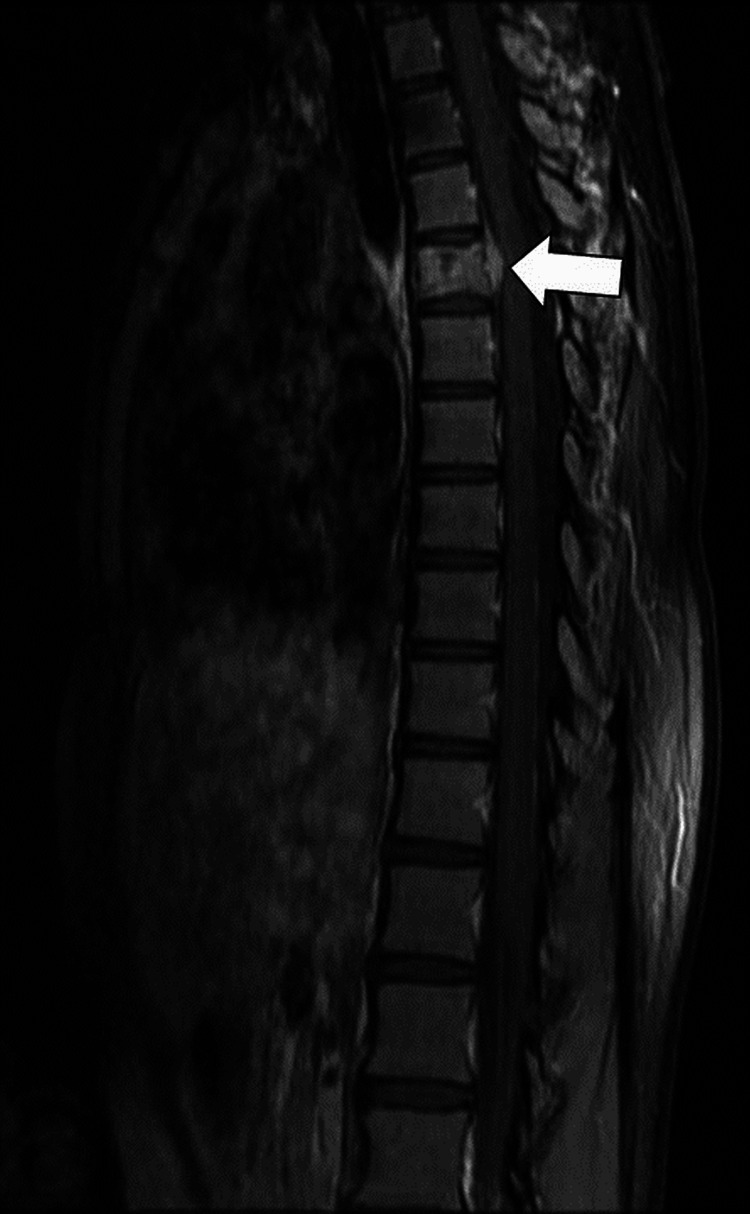
Saggital T2-weighted MRI shows a heterointense lesion (arrow) in the D5 vertebra with a significant reduction in vertebral body height consistent with vertebral metastasis.

It was found that there was mild anterior wedging of the vertebral body of the D5 vertebra along with a decrease in the height of the vertebrae (Figure 8), which suggests metastasis.

**Figure 9:**
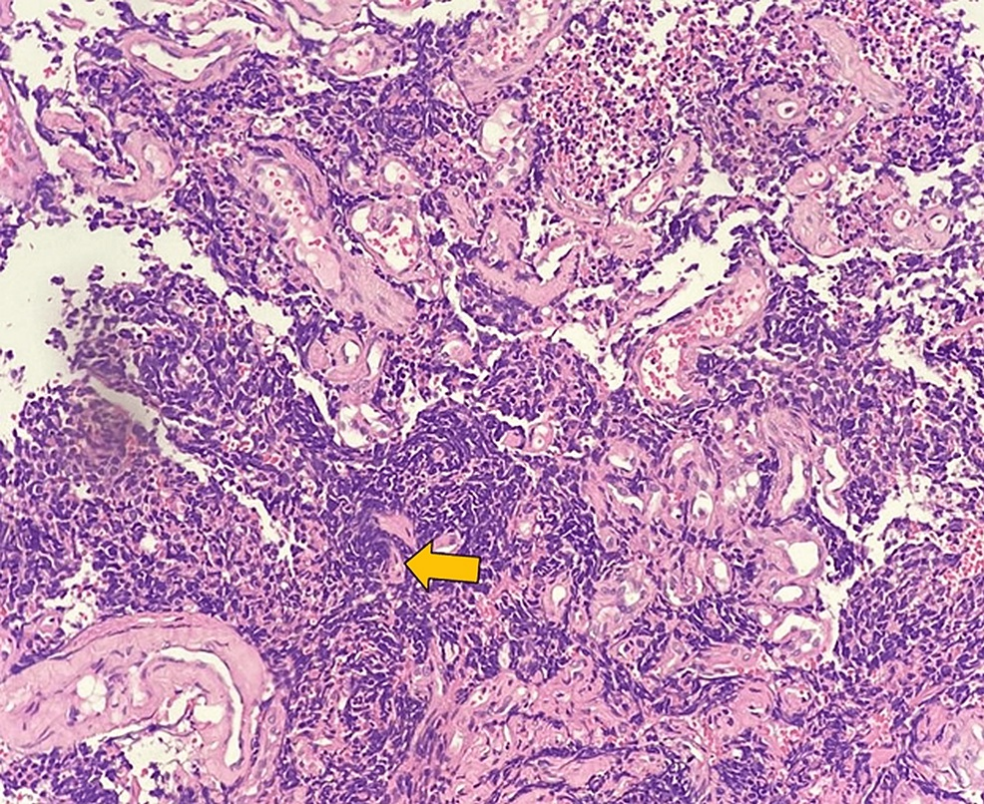
Immunohistochemistry Microscopy shows atypical small round blue cells arranged in pseudorosette (yellow arrow) and sheet-like arrangement with scant clear cytoplasm, round nucleus with finely stippled chromatin, and inconspicuous nucleoli.

Based on the imaging features described above and the patient's age, the patient was diagnosed with EES but also considered rhabdomyosarcoma as an alternative diagnosis. Additionally, the mass was subjected to core needle biopsy and immunohistochemistry, where clusters of atypical small blue round cells were observed [5] with scant clear cytoplasm and sheet-like arrangement in accordance with our imaging differentials (Figure 9). 

The tumor was found to be positive for cluster of differentiation (CD) 99, vimentin, and Friend leukemia integration 1 transcription factor (FLI1), and negative for leukocyte common antigen (LCA) and synaptophysin with a 7% Ki 67 positivity confirming the diagnosis of EES. As the Askin tumor (Figure 9) presents in the thoracic and pleural regions, this case represents a unique presentation of ESFT. 

Due to a lack of adequate research on EES, management was largely derived from soft tissue sarcoma protocol (SIOP); however, recent trials advocate better results following National Comprehensive Cancer Network (NCCN) recommendations on ES management to formulate a tailored approach to individual cases of EES based on presentation [6]. In accordance, our patient initially underwent four cycles of induction chemotherapy, each having alternate cycles of vincristine, doxorubicin, cyclophosphamide (VDC), and ifosfamide and etoposide (IE) with a three-week interval in between, thus each cycle lasted a period of six weeks. There was complete resolution of the soft tissue component with multidrug systemic chemotherapy. This was followed by surgical resection, but postoperative biopsy showed evidence of micrometastasis (R1 resection). Targeted adjuvant radiotherapy (45Gy) using a 3D conformal technique was employed for local control and satisfactory palliation was thus achieved. Currently, the patient is scheduled for a monthly follow-up assessment.

## Discussion

The incidence of EES is 0.4 per million [7]. In comparison with the classical skeletal variant, adults have a higher ratio of EES. The disease process appears to be unrelated to common risk factors of malignancies, including family history, radiation history, or environment. Patients with ESFT commonly complain of tenderness and localized swelling. When cancer progresses to a metastatic stage, symptoms include fever, weight loss, and back pain.

The MRI is a preferred method for assessing soft tissue involvement and disease staging. An MRI can assist in determining the relationship between a lesion and adjacent neurovascular structures, which helps plan biopsy and surgical resection. An EES typically exhibits intermediate to hypointense T1 intensity, hyperintense T2 intensity, and intense gadolinium enhancement. A CT scan can reveal intra-tumoral calcification (common in osteosarcoma and tuberculosis, however, uncommon in EES [1]), bony erosions, metastases, and lung involvement. Lesions attenuate similarly to muscles in CT. There were no signs of calcification in our imaging results.

Molecular genetics and immunohistochemistry techniques are required for the definitive diagnosis of the differentials that share common radiological imaging characteristics. Despite this, radiological imaging is essential for early detection, planning, and management. Other major differential diagnoses included embryonal rhabdomyosarcoma and lymphoma in this clinical case. Ewing's sarcoma is microscopically characterized by clusters of small round blue cells with a sheet-like arrangement or pseudo-rosette formation and scant, clear cytoplasm. Rhabdomyosarcoma and lymphoma also exhibit these characteristics. Further differentiation was achieved using immunohistochemical markers. In ESFT, periodic acid-Schiff (PAS) positivity and diastase sensitivity are observed, but not in lymphomas. Ewing’s sarcoma family of tumors is characterized by the presence of CD99, a membrane glycoprotein marker that is highly sensitive but not specific. Other positive markers include neural markers S-100 and synaptophysin, and vimentin. Friend leukemia integration is a relatively specific marker targeting transcription factors in the characteristic translocation of ESFT. The CD99, vimentin, and FLI 1 tests were positive within this case along with radiological findings that confirmed EES. The translocations t[11;22] [q24;q12] and t(21:22) (q22:q12) detected by molecular genetics [8,9] add additional value to the diagnosis of EES and ESFT. However, these were not performed due to the financial constraints of the patient.

This case report presents a young female patient presenting with a painless paravertebral and posterior chest wall mass coupled with pleural involvement and vertebral metastases. Askin tumors are ESFTs with thoraco-pulmonary involvement. Infrequently, this malignant neuroepithelioma occurs between the ages of 10 and 30 years, with a median age of 14.5 years and female predominance [10]. Our patient belongs to this stated age group (16 years) and gender. Our case, however, did not exhibit rib erosion or pleural effusion, which are common features of Askin (63% of cases) [11]. The presence of pulmonary involvement (less than 25%) and metastasis to bone or marrow (between 20% and 25%) has also been reported [11]. Because of its aggressive nature and higher tendency to recur, Askin tumor requires a precise diagnosis, which is difficult to make. 

Owing to lacunae in research specific to EES, management was derived from the soft tissue sarcoma protocol set by the Society of Pediatric Oncology (SIOP). Recent trials, however, advocate better results following NCCN's recommendation for ES to formulate a tailored approach to individual cases [6]. Broadly, management includes systemic and local treatment. Surgery is the mainstay of treatment for EES to achieve local control of the lesions (margins being 1 cm in bone, and 2 mm to 5 mm in soft tissue). Unresectable lesions near neurovascular structures may require radiotherapy with a dose of 54-55 Gy depending on the site involved [12]. Systemic chemotherapy reduces tumor size, eliminates micrometastases, and reduces recurrences [1]. 

The patient was administered systemic chemotherapy VDC and IE alternatively at three-week intervals, with each VDC/IE cycle lasting six weeks. Following each dose of dense VDC (75%)/IE (75%), pegylated granulocyte colony-stimulating factor (peg GCSF) (filgrastim) was given the following day to counter possible neutropenia. A total of four such cycles were completed in the induction phase during which the patient developed grade 2 chemotherapy-induced nausea and vomiting which settled with olanzapine and non-neutropenic fever which was managed conservatively with antibiotics. After chemotherapy, 18F-fluorodeoxyglucose (18F-FDG)-positron emission tomography (PET) CT scan revealed complete resolution of soft tissue mass, a decrease in marrow activity in the D5 vertebral body (standard unit value (SUV)max dropped from 4.89 to 3.47). Postoperatively, the biopsy showed evidence of tumor cells in the margin (R1 resection) likely due to micrometastasis. Thus, adjuvant targeted radiotherapy 45Gy in 25 fractions, 1.8Gy per fraction was delivered using a 3D conformal technique with 6mV photon between the D8 to D12 vertebra and satisfactory palliation was thus achieved. Currently, the patient is symptom-free and is scheduled for a monthly follow-up assessment. Furthermore, depending on the status, the patient is planned to undergo chemotherapy with the same multidrug regimen for a period of up to one year. Overall, in both ES and EES, metastatic disease carries a worse prognosis and poorer survival rates [13].

## Conclusions

When evaluating soft tissue neoplasms in children or adolescents, EES should be considered. Due to the poor prognosis of this condition, early detection is crucial. Diagnostic imaging, in particular MRI, is useful in detecting this disease as well as aiding in staging, and assessing response to therapy. It also assists in planning direct surgical resections, as well as recognizing local recurrences or metastases.
